# Editorial: The Fibroblast Growth Factor Signaling Pathway in Metabolic Regulation, Development, Disease, and Repair After Injury

**DOI:** 10.3389/fphar.2020.586654

**Published:** 2020-09-08

**Authors:** Zhouguang Wang, Li Lin, Jin-San Zhang, Xingxing Zhong, Saverio Bellusci, Xiaokun Li

**Affiliations:** ^1^ School of Pharmaceutical Sciences, Wenzhou Medical University, Wenzhou, China; ^2^ Department of Molecular Pharmacology, Albert Einstein College of Medicine, Bronx, NY, United States; ^3^ Division of Oncology Research, Mayo Clinic, Rochester, MN, United States; ^4^ Cardiopulmonary Institute, Member of the German Lung Center, Justus Liebig University, Giessen, Germany

**Keywords:** FGF (fibroblast growth factor), regeneration, development, metabolic regulation, ischema-reperfusion injury

Fibroblast growth factors, or FGFs, are a family of structurally related proteins with diverse functions during embryonic development, tissue repair, cancer, and metabolic homeostasis. In humans, 22 members of the FGFs family have been identified, all of which are structurally related signaling molecules. They have been alternately referred to as “pluripotent” growth factors and as “promiscuous” growth factors due to their multiple actions on a wide range of cell types. Four receptor subtypes of FGFs can be activated by more than 20 different FGF ligands. Thus, the functions of FGFs in developmental processes can include mesoderm induction, anterior-posterior patterning, limb development, neural induction, and neural development.

This Research Topic gathers original research and review papers on the different roles of FGFs/FGFRs in early development, organogenesis, musculoskeletal biology, nervous system, metabolism, tumorigenesis, intracellular signaling, and emerging research areas. This collection of papers sheds light on the drug development of FGF, with a focus on the new development for FGFs treatment or mechanisms of action, ranging from basic research to clinical translational studies.

The 17 accepted articles consist of 14 Original Research articles and 3 Reviews or Mini-Reviews, which demonstrated roles of FGFs in various diseases including neural diseases, diabetes-related diseases, renal injury, lung diseases, digestive diseases, and vascular diseases.

Four research articles elucidated that FGFs are involved in alleviating neural diseases *via* different mechanisms. By using 6-OHDA–induced Parkinson’s disease (PD) mice model, Zhong et al. found that acid FGF promoted autophagy by inhibiting ER stress-induced TRB3 overexpression during PD development and subsequently ameliorated 6-OHDA–induced neuronal apoptosis (Zhong et al.). Zhu S. et al. reported the anti-apoptotic effect of another FGF member FGF22 in mediating neural disease (Zhu S. et al.). FGF22 treatment was associated with reduced pro-apoptosis proteins and increased recovery of the spinal cord injury in mouse animals. Intriguingly, the number of neurons and expression of an axon regeneration related protein (growth-associated protein 43) were also increased after FGF22 administration. The beneficial effects of FGF22 in ER stress-induced spinal cord injury could be partially due to neuron regeneration (Zhu S. et al.). This hypothesis is consistent with what has been claimed by Dong et al. that FGF10 treatment promoted axonal regeneration and functional recovery in sciatic nerve injury rat (Dong et al.). FGF10 was also revealed to prevent Schwann cells from oxidative stress-induced apoptosis, which was probably related to the activation of phosphatidylinositol-3 kinase/protein kinase B (PI3K/Akt) signaling (Dong et al.). In addition to the roles of anti-apoptosis and promoting regeneration, the anti-inflammatory function of FGFs were also involved in repairing neural diseases. Wang et al. treated LPS-induced depression mouse model with recombinant human FGF2 and found that depressive-like behavior was significantly relieved (Wang et al.). The decreased microglial expression of proinflammatory cytokines suggested the involvement of FGF2 in NF-κB suppression (Wang et al.).

Four of these research articles investigated roles of FGFs in diabetes-related diseases, providing new insights into treatment of diabetes and related complications. In Xu et al.’s research, db/db mice revealed improved blood glucose level and diabetes-induced liver steatosis, fibrosis and apoptosis after intraperitoneally injected with FGF1 (Xu et al.). Mechanistic investigations suggested that these effects were the results of attenuated oxidative stress and ER stress (Xu et al.). The anti-oxidative stress function of another FGF member, basic Fibroblast Growth Factor, was shown to play vital roles in ameliorating diabetic nephropathy (Wei et al.). Studies have shown that FGF1 has a wide range of physiological functions, the application *in vivo* is limited because of the lack of an efficient and safe delivery system. Nanoliposomes and ultrasound targeted microbubble destruction techniques provided hopes into solving this problem. Zheng et al. evaluated the preventive effect of FGF1-loaded nanoliposomes (FGF1-nlip) combined with ultrasound-targeted microbubble destruction (UTMD) on diabetic cardiomyopathy using ultrasound examination and found that echocardiographic indexes were significantly higher than those in FGF1 and FGF1-nlip treatment groups (Zheng et al.). Islet transplantation is considered a potential therapy for diabetes. However, the extracellular matrix (EXM) proteins essential for islets survival are impaired in the isolation process before islet transplantation. Zhu Q. et al. elucidated the beneficial effects of recombinant human collagen with FGF2 application in islet transplantation. The system provided insights into islet transplantation with a simulated EXM microenvironment for the revascularization and attachment of islets to the transplantation region (Zhu Q. et al.).

On another hand, Tan et al. investigated the molecular pathways underlying the protective effect of FGF10 on renal ischemia-reperfusion (I/R) injury using Sprague-Dawley rat model (Tan et al.). It was shown that FGF10 attenuated I/R-induced renal epithelial apoptosis by suppressing excessive ER stress in renal I/R injury, and the function was partially mediated by the activation of the MEK–ERK1/2 signaling pathway. Accumulating evidence suggests that FGFs play important roles in renal I/R injury. As summarized in Deng et al.‘s review (Deng et al.), FGFs mediate repair process of I/R injury-caused acute kidney injury and could offer a potential therapeutic option in the future. Chen Q. et al. studied the physiological role of FGF21 in cisplatin-treated AKI and it was revealed that recombinant FGF21 significantly improved renal function in cisplatin-induced damage *via* SITT1 signaling pathway (Chen Q. et al.). Non-mitogenic FGF1 was found to enhance angiogenesis following ischemic stroke by regulating the sphingosine-1-phosphate 1 pathway (Zou et al.). Two reviews summarized the progress of FGF in corneal neovascularization (Chen M. et al.) and fibroblast FGF10 in duodenal atresia (Jones et al.).

In addition to neural diseases, diabetes-related diseases and renal damage, functions of FGFs were also investigated in other diseases. It was claimed by Lin et al. that administration of engineered FGF1 mutant-FGF1^ΔHBS^ protected liver in alpha naphthylisothiocyanate (ANIT)-induced intrahepatic cholestasis mice by reducing hepatic bile acid accumulation (Lin et al.). Yuan et al. found that the temporospatial expression of FGFR1 and FGFR2 varied during lung development, homeostasis, and regeneration, indicating the involvement of FGF signaling pathways in lung development and diseases (Yuan et al.). To overcome the treatment resistance toward problem of the tyrosine kinase inhibitor (TKI) in non-small-cell lung cancer (NSCLC), Chen G. et al. evaluated the efficacy of the compound 15c, a novel dual inhibitor of EGFR^L858R/T790M^ and FGFR1, which revealed that the compound 15c efficiently overcame the EGFR-TKI resistance of NSCLC (Chen G. et al.).

In summary, both the research articles and reviews in this Research Topic are an excellent source of information about the current knowledge in the fibroblast growth factor signaling pathway in metabolic regulation, development, disease, and repair after injury field.

## Author Contributions

ZW and XZ wrote this article. LL, J-SZ, SB and XL have made a direct and intellectual contribution to the work. All authors have approved the article for publication.

## Funding

This work was supported by grants from Advanced Postdoctoral Programs of Zhejiang (zj2019030 to Z.W.), China Postdoctoral Science Foundation (2019M662015 to Z.W.). National Natural Science Foundation of China (No.81771284, 81971180 to L.L.), Research Unit of Research and Clinical Translation of Cell Growth Factors and Diseases, Chinese Academy of Medical Science (No.2019RU010 to X.L.).

## Conflict of Interest

The authors declare that the research was conducted in the absence of any commercial or financial relationships that could be construed as a potential conflict of interest.

